# Supplementary material for: Shared neural representation between motor execution and kinesthetic imagery in the primary motor cortex
Source: Imaging Neurosci (Camb). 2026 Feb 17;4:IMAG.a.1141. doi: 10.1162/IMAG.a.1141 (PMC12915000; doi:10.1162/IMAG.a.1141)
Supplement: Supplementary Material [file IMAG.a.1141_supp.pdf]

Supplementary materials

EMG recording and processing

The electromyography (EMG) was measured by BrainAmp ExG MR (BRAIN PRODUCTS) and recorded with the Recorder (BRAIN PRODUCTS) with a sampling rate of 5000 Hz, low cutoff and DC, high cutoff of 1000 Hz. During EMG recording, the EMG clock was synchronized with the clock of an MRI scanner. Offline processing (e.g., noise filtering) of the EMG data was done using Analyzer2 (BRAIN PRODUCTS) in the following order: 1) MR noises were excluded, 2) EMG data were processed using the IIR (zero phase-shift Butterworth) filter with low cutoff: 5 Hz; high cutoff: 100 Hz; Notch: 50 Hz; Bandreject: 17, 33, 50, 67 Hz; bandwidth: 4 Hz, 3) EMG data were downsampled from 5000 Hz to 500 Hz, and 4) EMG data were rectified.

As written in the EMG analysis of results, the analyzed EMG data were extracted from the task blocks of the execution and imagery sessions, and the corresponding timing of the fixation session as a rest period. Generally, some participants can relax well when task onset comes, but others cannot. Cared about such an individual difference, each EMG data extracted from the target block was standardized by the standard deviation of the EMG values that were obtained from the 300 ms period just before each target block.

Result of the functional localizer

Supplementary Table 1: Anatomical regions, coordinates of peak voxel, and *t*-values of observed activations in the functional localizer of the first study.

Anatomic region	voxels	MNI coordinates			*t*-value
		x	y	z	
R BA18	539	6	-64	-16	15.49
R BA37		24	-49	-25	14.63
R BA37		21	-55	-19	12.08
L BA4	714	-39	-22	62	15.16
L BA3		-33	-34	62	13.91
L BA3		-45	-31	59	12.69
L BA48	181	-60	-16	11	13.39
L BA41		-48	-31	17	11.23
L BA48		-54	-19	20	7.17
L BA19	140	-27	-67	-22	10.08
L BA18		-18	-85	-16	9.02
L BA18		-15	-76	-19	5.34

MNI, Montreal Neurological Institute; L, left hemisphere; BA, Brodmann Area.

Results of the mass-univariate group analysis (Figure caption)

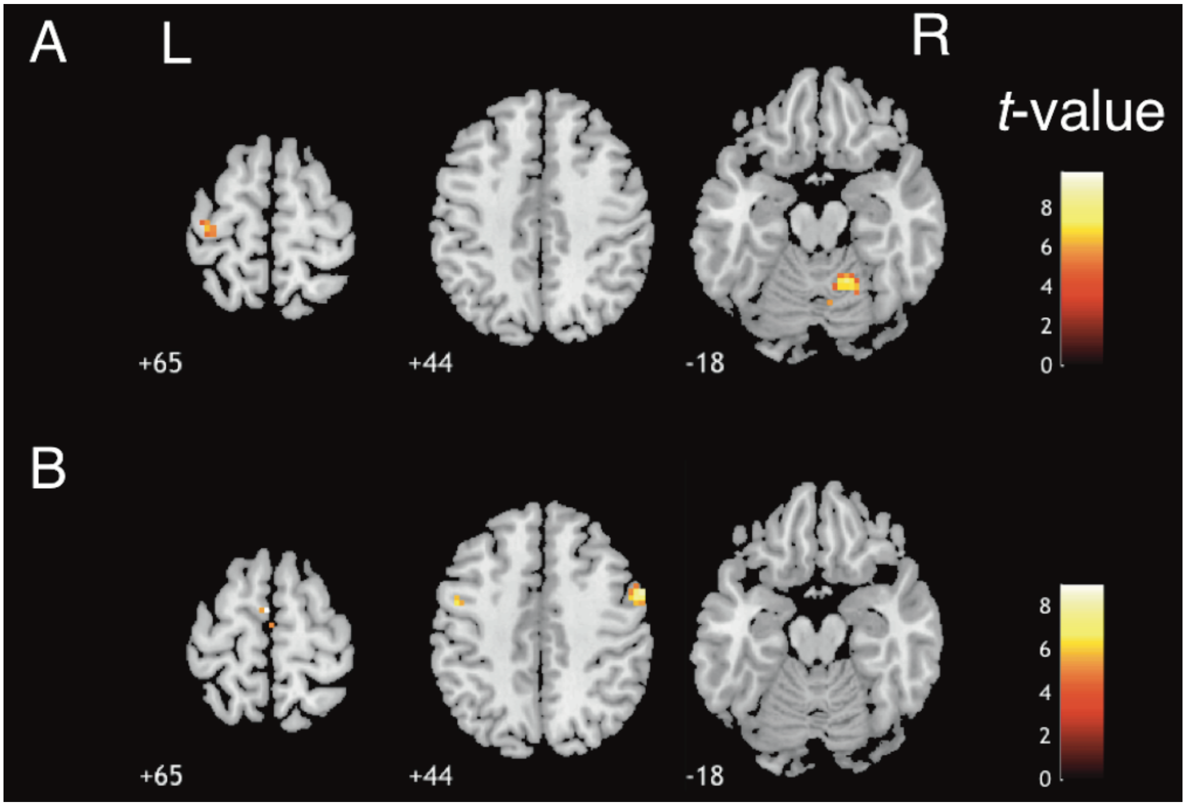

Supplementary Figure 1:

(A) The results of mass-univariate group analysis for the execution task session of the first study, with an uncorrected threshold level of $k \geq 10$ ($p < 0.0005$). The detected left cluster, consisting of BA4 (i.e., X = −39, Y = −19, Z = +65, $t = 4.86$) and BA6, is indicated by the colored area. The detection of BA4 was to be expected, because this region relates to motor control of the contralateral body parts (e.g., Heming et al., 2019). (B) The results of mass-univariate group analysis for the imagery task session in the original study with an uncorrected threshold level of $k \geq 10$ ($p < 0.0005$). The detected left cluster included BA6 (i.e., X = −3, Y = −4, Z = +65, $t = 8.98$) and is indicated by the colored area, but no voxels

from BA4 were detected. SMA is frequently reported as an active region during motor imagery (e.g., Hanakawa, 2016), while M1 activation is rarely reported (Hétu et al., 2013). These results correspond to those in previous studies.

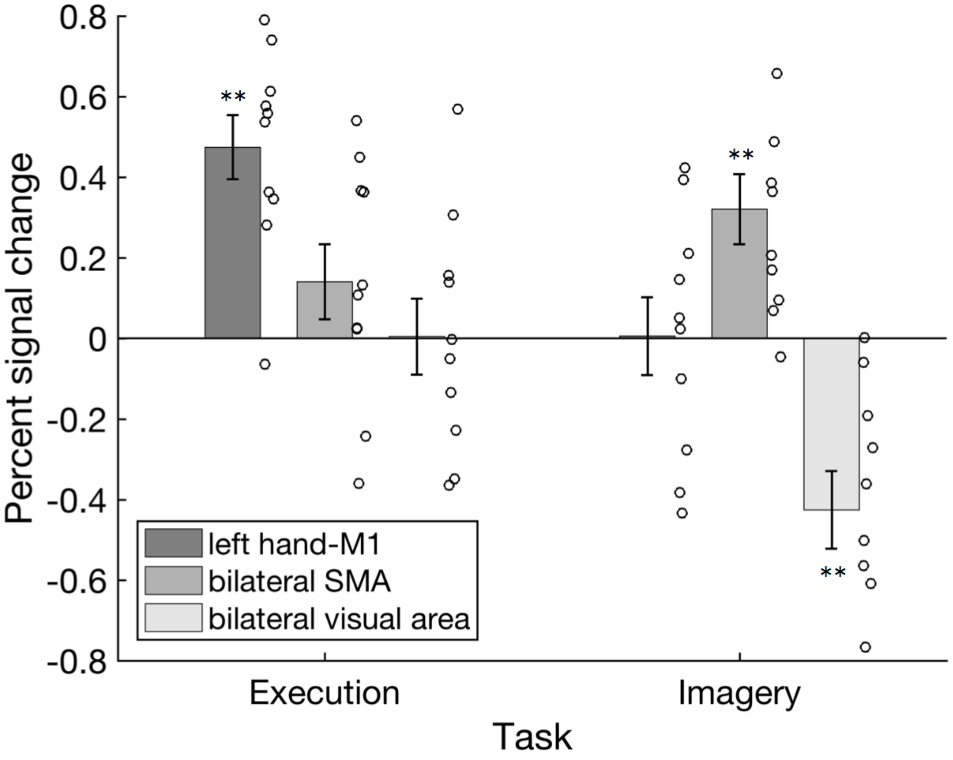

Supplementary Figure 2:

The result of the mass-univariate analysis with modeling of rest blocks: The average signal-change rate of each ROI in each task session. Error bars show standard errors. Circles indicate single data. **: $p < 0.01$. Consistent with the result of the mass-univariate analysis without modeling rest, significant differences from rest were found at three locations. Left hand-M1 in motor execution task (t (9) = 5.97, $p < 0.01$ (two-tailed), d = 1.99), bilateral SMA (t (9) = 3.7, $p < 0.01$, d = 1.23), and bilateral visual area (t (9) = -4.43, $p < 0.01$, d = 1.48) in motor imagery task. These results supported our conclusion based on the result of the mass-univariate analysis with modeling rest.

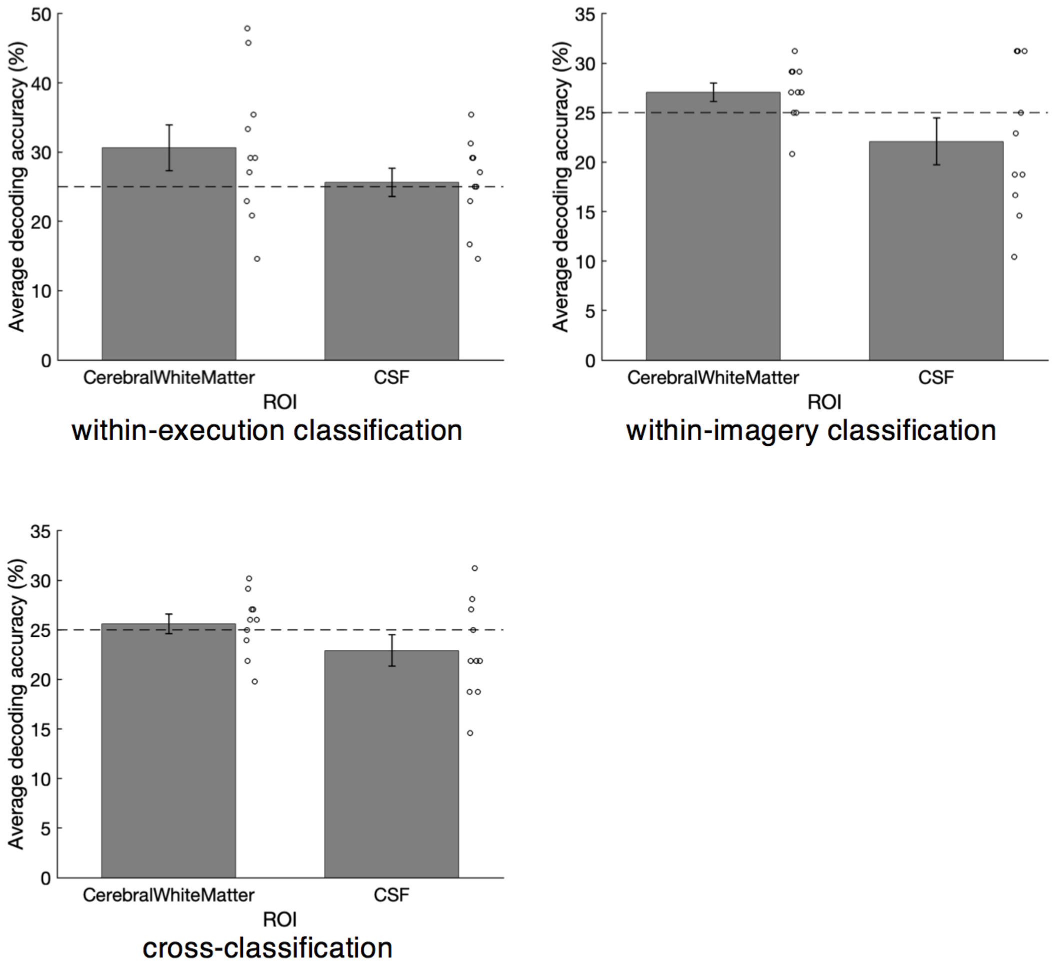

Supplementary Figure 3:

The average decoding accuracy of two control ROIs (cerebral white matter and CSF) in our three main classifications, i.e., within-execution classification (top left), within-imagery classification (top right), and cross-classification (bottom left). Error bars are standard errors. The horizontal dashed line indicates the chance level (25%). Circles indicate single data. In each case, there was no significant decoding accuracy compared to the chance level ($|t\text{s}\ (9)| < 2.24$, $p\text{s} > 0.05$ (two-tailed), $d\text{s} < 0.75$). Therefore, the significant results of MVPA in the left hand-M1 are unlikely to be by chance.

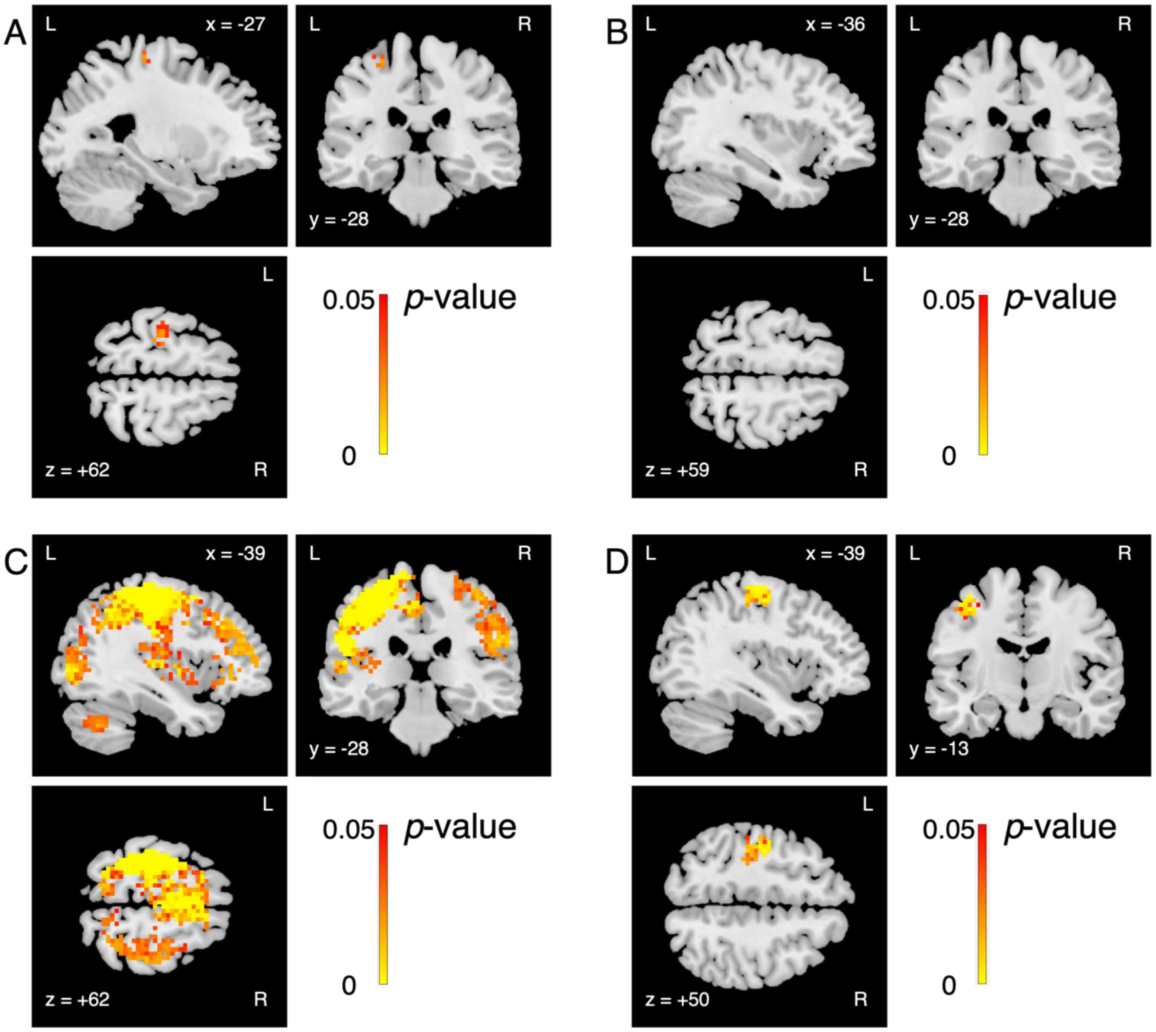

Supplementary Figure 4:

The FWE/TFCE-corrected versions of the searchlight analyses were performed with the following parameters: number of permutations = 10000; H = 2; E = 0.5; C = 6; dh = 0.1, and corrected $p < 0.05$: A) within-execution classification, B) cross-classification, and C) between-modality classification in the first study. D) cross-classification in the replication study. Similar to the uncorrected searchlight results (see Figure 3E and 5B), these analyses detected a cluster including BA4 voxels, despite no cluster being detected in the first study with cross-classification. Note that these FWE/TFCE-corrected searchlights were more

conservative than the uncorrected thresholds accepted in Figure 3E and 5B ($p < 0.0005$, $k \geq 10$).

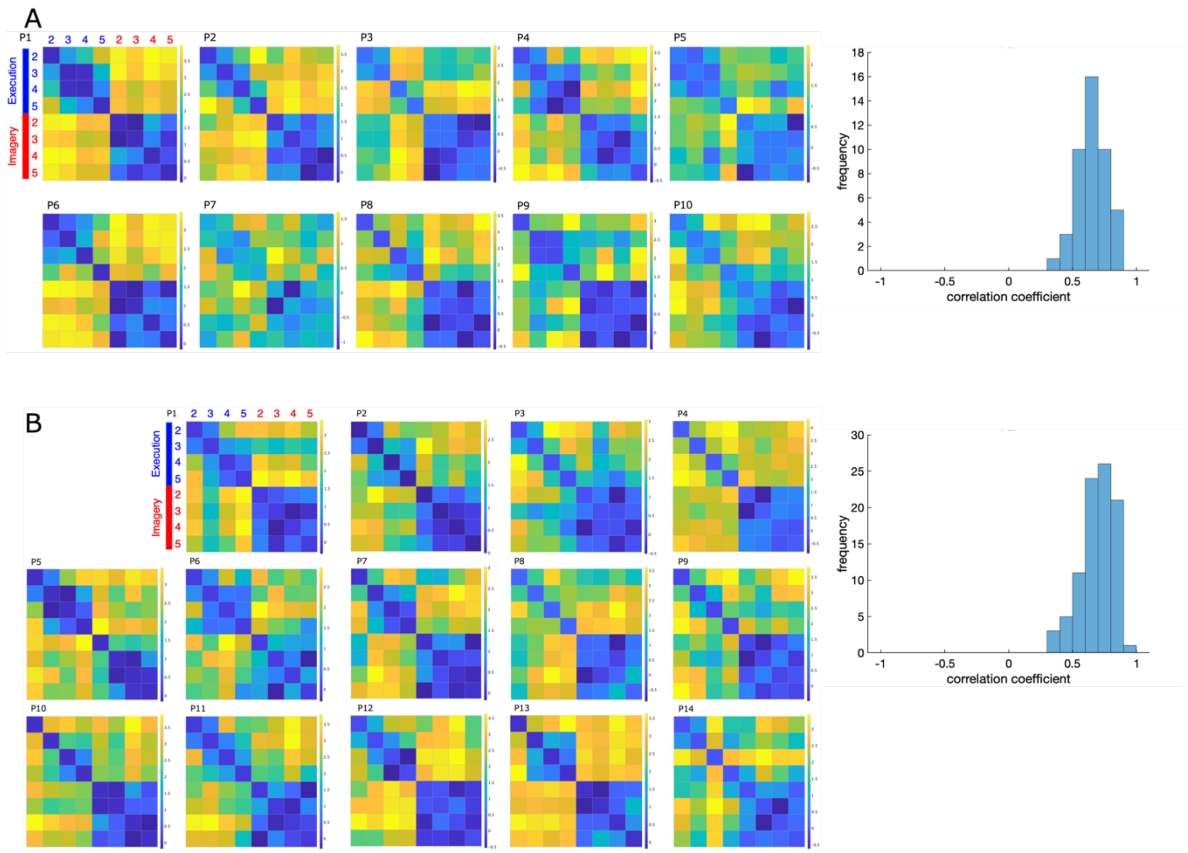

Supplementary Figure 5:

RDM of each participant in A) the first study and B) the replication study. The right-hand histogram for each study shows the frequency distribution of the correlation coefficients between individual RDMs across participants. These correlations were calculated using the values of the non-diagonal cells in individual RDMs.

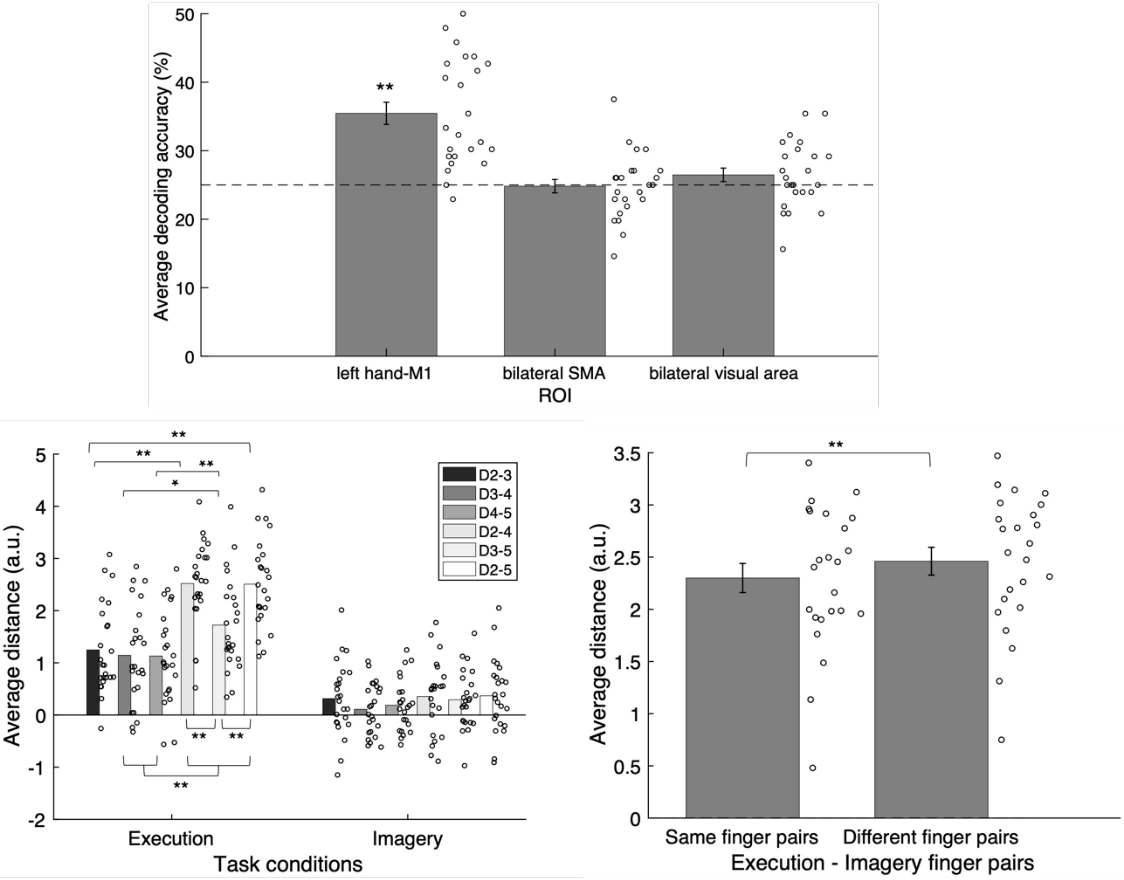

Supplementary Figure 6:

The pooled data results of cross-classification and distance analysis. Top figure showed the results of one-sample *t* tests for respective ROIs with the first and the replication studies (n = 24). Similar to the results of both studies, only left hand-M1 showed significant highly chance accuracy (mean = 35.5, SD = 7.91, t (23) = 6.48, d = 1.35, p < 0.0001; for the bilateral SMA and visual areas, means (SDs) were 24.83% (4.775) and 26.48% (4.84), respectively, |*t*s| (23) < 1.50, *d*s < 0.31, *p*s > 0.149. Bottom left figure indicated the result of a two-way within ANOVA (two tasks × six finger pairs) on the distance of the finger pairs with n = 24 (see RDM in Figure 6). The results were similar to those of the first and

replication study. Both the main effects of task and finger pair were significant (F (1, 23) = 83.94, $p < 0.001$, ${}_{p}\eta^2 = 0.79$, F (5, 115) = 18.94, $p < 0.001$, ${}_{p}\eta^2 = 0.45$, respectively). The interaction was also significant (F (5, 115) = 11.28, $p < 0.001$, ${}_{p}\eta^2 = 0.33$), and post hoc analysis showed the significant simple main effects of the task on each finger pair (Fs (1, 138) > 17.54, ps < 0.001), with all the execution finger pairs having a greater distance than the imagery finger pairs. A significant simple main effect of the finger pair on the execution task was also observed (F (5, 230) = 29.41, $p < 0.001$), whereas the simple main effect of the finger pair on the imagery task was not significant (F (5, 230) = 0.70, $p = 0.62$). Multiple comparison tests using Tukey's honestly significant difference showed that each pair of adjacent fingers had a smaller distance than the index-ring finger pair and the index-little finger pair in the execution task. Furthermore, the ring-little finger pair and middle-ring finger pair had a smaller distance than the middle-little finger pair (ds > 0.61, ps < .05). The bottom right figure showed the respective mean distances for the same (diagonal) finger pairs and different (nondiagonal) finger pairs in the region of the execution-imagery combination in the RDM in Figure 6 (n = 24). A two-way paired t-test showed that the mean distance (SD) of the same finger pairs (2.30 (0.68)) was significantly shorter than that of the different finger pairs (2.46 (0.65)) (t (23) = -4.85, $d = 1.01$, $p < 0.001$). Although these results and figures are natural, they indicate consistency of the findings in the initial and replication studies. In all these figures, *: $p < 0.05$, **: $p < 0.01$.
